# Supplementary figures and images for: A high-density genetic map and molecular sex-typing assay for gerbils
Source: Mamm Genome. 2019 Apr 10;30(3):63–70. doi: 10.1007/s00335-019-09799-z (PMC6491409; doi:10.1007/s00335-019-09799-z)

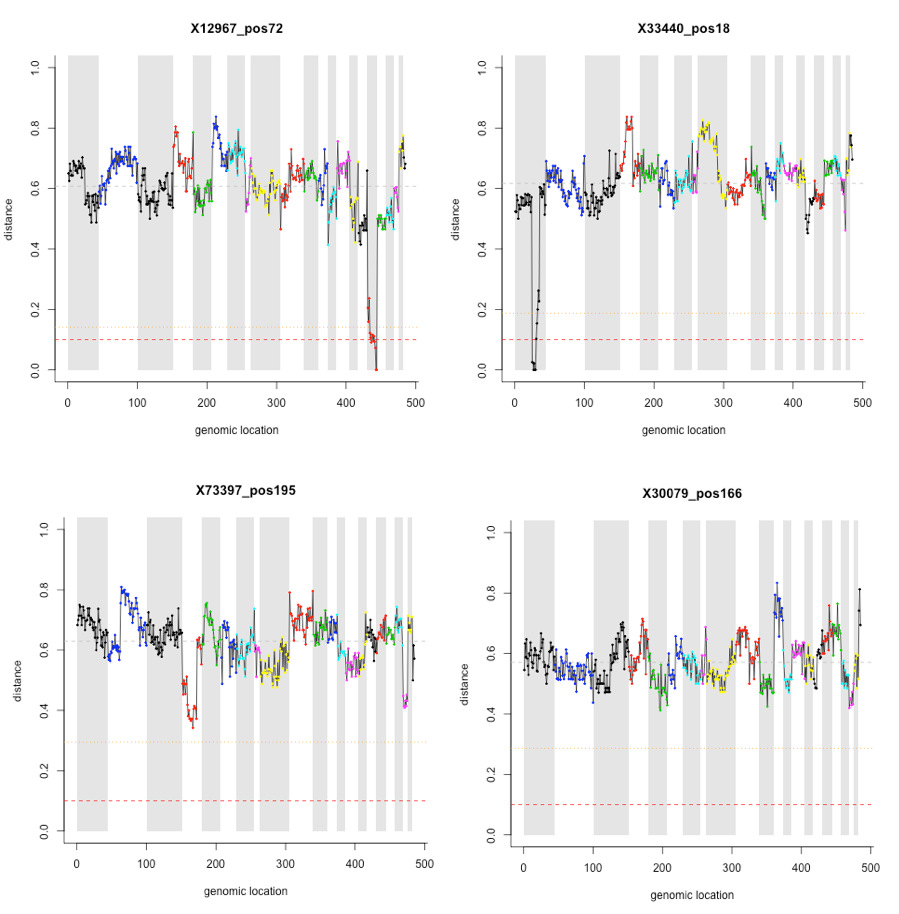

Supplement: Supplementary file 2 — Online Resource 2: Placing additional markers on the map. Two examples of markers with strong association at specific locations on the map (top row: 12967_pos72 and 33440_pos18) and two examples of markers without a strong association (bottom row: 73397_pos195 and 30079_pos166). The X-axis is the ordered set of the 485 high-quality markers that comprise the map. Linkage groups are differentiated by point colour and alternating grey background stripes. The Y-axis is the genotype distance between two markers, measured as the proportion of mismatching genotypes in the comparison between the unplaced marker and each map location. The grey dashed line is the genome- wide average, the orange dotted line is 4 standard deviations away from the mean, and the red dashed line is the 10% cutoff. Markers were only associated with the map if they had fewer that 10% mismatching genotypes, in which case they were placed at the same centiMorgans as the first marker with which they shared the most matching genotypes (PNG 209 kb) [file 335_2019_9799_MOESM2_ESM.png]

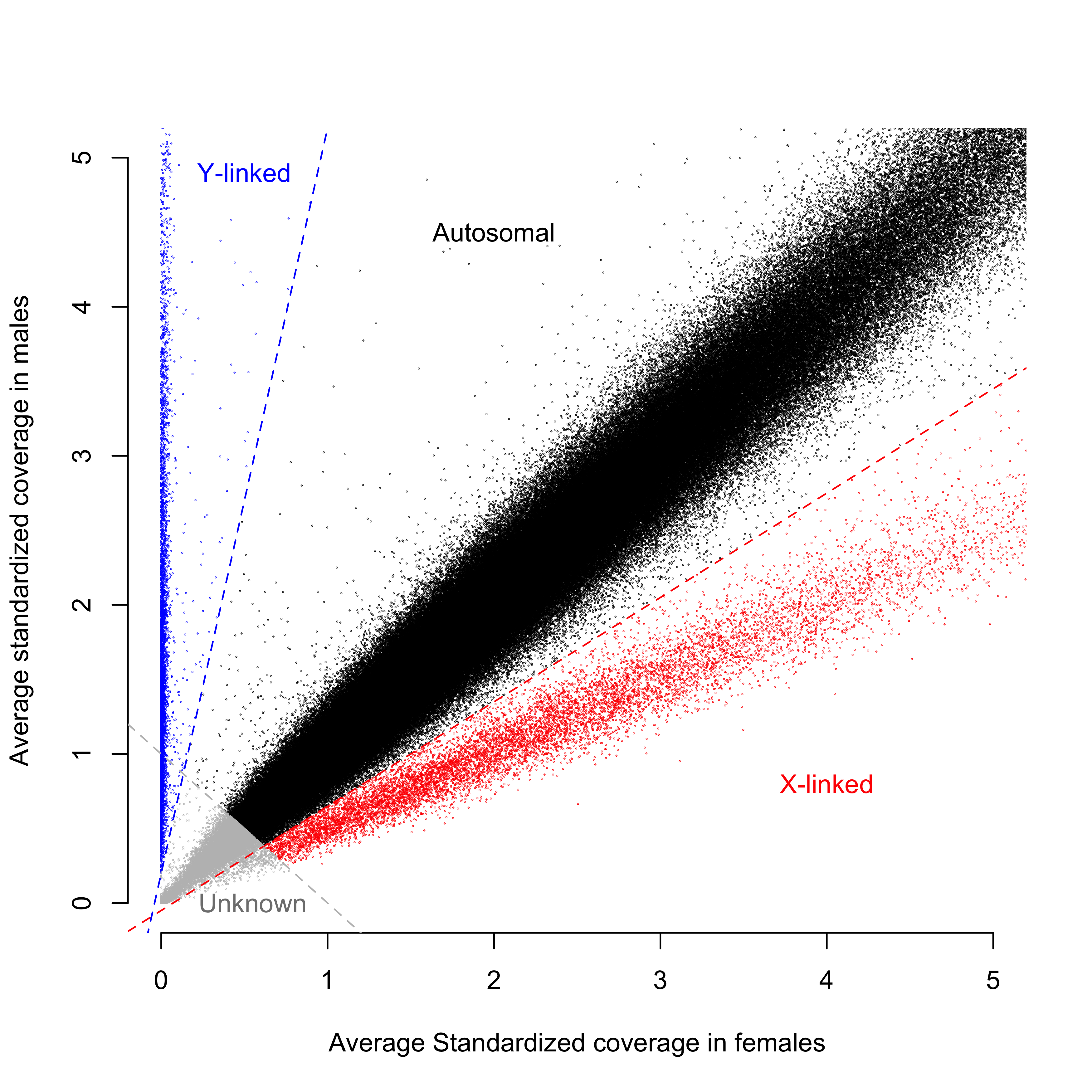

Supplement: Supplementary file 3 — Online Resource 3: Boundaries for annotating sex-linkage in markers. This shows an extreme close-up of the cutoffs chosen to differentiate Autosomal, X, and Y linked markers. The blue line distinguishing Y-linked markers is at: coverageMale = (5 ∗ coverageFemale) + 0.2, the grey line to identify unknown markers is at coverageMale = 1 − coverageFemale, and the red line to distinguish X-linked markers is coverageMale = (7 / 10 ∗ coverageFemale) − 0.05.These cutoffs were chosen by visually inspecting this plot for the natural breakpoints between Autosomal-, X-, and Y-linked makers with the observation that Y-linked markers cluster at coverageFemale = 0, Autosomal markers cluster at coverageMale = coverageFemale, and X-linked markers cluster at coverageMale = 1/2 8 coverageFemale. The boundary for the unknown markers is set conservatively to avoid mistaking low-coverage X-linked and Autosomal markers (PNG 2901 kb) [file 335_2019_9799_MOESM3_ESM.png]
